# Antecedents to the Adoption of Mobile Payment in China and Italy: an Integration of UTAUT2 and Innovation Resistance Theory

**DOI:** 10.1007/s10796-021-10237-2

**Published:** 2022-01-22

**Authors:** Giacomo Migliore, Ralf Wagner, Felipe Schneider Cechella, Francisco Liébana-Cabanillas

**Affiliations:** 1 Università di Trento, Trento, Italy; 2 University of Kassel, Kassel, Germany; 3 Universidad de Granada, Granada, Spain

**Keywords:** Mobile payment, UTAUT2, Innovation resistance theory, China, Italy, Risk barriers

## Abstract

This research aims to investigate the adoption gap in mobile payment systems between Italy and China, focusing on users’ intention to adopt mobile payment. The theoretical framing considers both drivers and barriers when combines the unified theory of acceptance and use of technology 2 (UTAUT2) with innovation resistance theory (IRT). To empirically verify the proposed model, this study gathers primary data through a web-based, self-administered survey. To analyze the data, we use structural equation modeling, and to test for significant differences between the two groups we run multi-group analysis. The respondents in Italy and China present different behaviors. Social influence plays a significant role in cultures with high uncertainty avoidance, such as Italy. The tradition barrier is the only significant barrier to the adoption of mobile payment.

## Introduction

The *Financial Times* devoted a special report to the future of payment in October 2020,^1^ central banks in China^2^ and Europe^3^ plan to launch digital versions of their currencies in the coming years, and COVID-19 has triggered a rapid surge in the portion of card and contactless payments in face-to-face purchases. Mobile payment combines contactless payment systems with mobile devices, enabling users to initiate, authorize, and complete financial transactions that move money through mobile networks or wireless communication technologies (Chandra et al., 2010; Lu et al., 2011; Oliveira et al., 2016).

In recent years, mobile payment systems have not been adopted equally quickly in various geographic locations. Notably, the European market generally appears to be slower to integrate mobile payment than other economies, despite a large degree of heterogeneity on the continent (Deloitte, 2019). Although the share of the population connected to mobile internet is greater in Europe than in the Asia-Pacific region (68% and 56%, respectively) (GSMA, 2019), only 31% of Europeans mentioned using mobile payment services in the previous month as compared to 37% of Asians (Global Web Index, 2019).

This study limited its research scope to mobile payment adoption in two countries: China and Italy. China is an attractive setting as it had the highest adoption rate of mobile payment worldwide in 2019 (eMarketer, 2019). Despite this already high adoption rate, opportunities remain for the implementation of new features in mobile payment in China. Also, the dynamism of the Chinese market requires constant investigation of the factors influencing the adoption of mobile payment (Miao & Jayakar, 2016). In Italy, the number of daily cash transactions per person is among the highest in Europe, while the use of digital payments is among the lowest (European Central Bank, 2017). Empirical research has highlighted the need to investigate the barriers that retard the adoption of mobile payment technologies (Moroni et al., 2015).

The mobile payment penetration rate among Chinese smartphone users is 81.1% as opposed to 21.1% among Italian users (eMarketer, 2019), so the contrast in mobile payment adoption between the countries is even starker than that between the European and Asian markets as a whole. Previous research suggests possible reasons for this difference. Notably, Chinese society puts greater emphasis on economic and technological progress as a means of achieving and maintaining the nation’s leadership position; at the personal level, it establishes an individual’s position within the hierarchical structure of the society (Qi Dong, 2009). By contrast, Italian society faces less political pressure regarding technology adoption. In their study comparing Italy’s and China’s e-commerce adoption, Capece et al. (2013) identified lack of trust and uncertainty avoidance as potential barriers in Italy in contrast to a stronger aspiration to progress and improvement in China. The influence of culture on the differing adoption of technologies between countries has also been widely investigated. Hofstede’s (2001) research and subsequent contributions building upon it studies the influence of cultural dimensions on the classic models of technology adoption. These dimensions of cultural influence have been analyzed in diverse contexts and countries, yielding important conclusions that explain cultural differences in technological contexts (Alcántara et al., 2018). However, cross-cultural studies on mobile payment adoption using Hofstede’s cultural dimensions remain scarce. To the authors’ knowledge, there is still no comparative study of mobile payment adoption comparing China and a European country.

This research investigated the reasons for the mobile payment adoption gap between China and Italy by analyzing individuals’ propensity and resistance to technology adoption. The study implements a model that includes both drivers and barriers to adoption (Zhou, 2013; Gao & Waechter, 2017). Accordingly, the conceptual framework draws upon two complementary theories: the unified theory of acceptance and use of technology 2 (UTAUT2) (Venkatesh et al., 2012) and innovation resistance theory (IRT) (Ram, 1987; Ram & Sheth, 1989). The resulting model was empirically tested with primary data retrieved from a web-based, self-administered survey.

The contribution of this research is threefold. First, it addresses a gap in the recent scholarly literature by integrating UTAUT2 and IRT (Leong et al., 2020; Tamilmani et al., 2021). Notably, Lin et al. (2019) and, more recently, Kaur et al. (2020) called for further investigation of the factors contributing to consumer resistance in the adoption of mobile payment and used IRT to frame the study of mobile payment adoption. However, IRT has not been considered as an explanatory framework in a cross-country comparison of mobile payment adoption yet. Second, an analysis of the results of this study suggests that IRT may not be suitable to identify and investigate the current barriers to the adoption of mobile payment in cross-cultural studies. Third, we contribute through the distinct findings for Italy and China; social influence plays a significant role in cultures with high uncertainty avoidance, and the tradition barrier is the only significant barrier to the adoption of mobile payment.

Section 2 of this article defines the key terms used in the research, summarizes previous relevant research, and outlines the theoretical framework. The proposed research model and hypotheses are introduced in Section 3, and Section 4 describes the data collection methodology, questionnaire design, and analysis. Sections 5, 6, and 7 provide, respectively, the results of the analysis, the main findings and discussion, including limitations and suggestions for future research.

## Theoretical Framework: Consumer Mobile Payment

Baron et al. (2006) emphasize that technology adoption is propelled by two components: (1) embedding the technology in the society and (2) acceptance by potential users. The first component is addressed in this study’s design by the antecedents of image, tradition, risk, and value barriers in the IRT framework, and the second is reflected in the classical UTAUT2 antecedents capturing individual motives and the amplifiers of technology adoption.

Mobile payment is an activity performed on an electronic device connected to the mobile internet that enables the completion of a financial transaction (Liébana-Cabanillas et al., 2014, 2020). From the consumer perspective, mobile payment refers to “all payments for goods, services, and bills authorized, initiated, or realized with a mobile device” (Schierz et al., 2010, p. 210). Regarding the device, mobile payment refers to transactions to acquire goods and services or make payments conducted on a cell phone, smartphone, or personal digital assistant using wireless communication technology (Singh et al., 2020; Dahlberg et al., 2008). Based on these definitions, this study defines mobile payment as all payments carried out by consumers through an application on a mobile device (rather than using cash, checks, or bank cards).

### Previous Research on Mobile Payment Adoption

Researchers began investigating the adoption of mobile payment in the early 2000 s. Seminal studies focused on connection protocols and on the main drawbacks to mobile payment (Wang et al., 1998; Kreyer et al., 2002; Hassinen et al., 2006) as well as on the interconnectivity of mobile payment systems and on their individual adoptions (Chen, 2008; Mezgec et al., 2008). The first proposals on the barriers and drivers of mobile payment adoption were made in Mallat’s qualitative study in 2007.

Based on this first round of studies, Dahlberg et al. (2008) conducted a review of the literature on mobile payment systems, analyzing the various factors affecting mobile payment services and suggesting directions for future research. In this context, mobile payment referred to the use by individuals of mobile devices, including wireless phones, personal digital assistants, radio frequency devices, and near field communication–based devices (NFC), to make payments for goods and services (Alkhowaiter, 2020; Patil et al., 2020). Several studies have analyzed the intention to use various mobile payment systems. Among the outstanding research are the studies on the use of short message service (SMS) as a mobile payment system (Liébana-Cabanillas et al., 2014), on NFC technology (Brumercikova & Bukova, 2020), and on quick response codes (QRs) (Gao et al., 2018), as well as on P2P payment (Liébana-Cabanillas et al., 2021), biometric payment (Pal et al., 2017), and even wearables (Singh & Sinha, 2020).

Mobile payment adoption has been widely investigated in various countries. Fourteen studies have analyzed mobile payment adoption in European countries and 19 in greater China (Liu et al., 2019), but cross-country comparison emerged only recently. Mobile adoption in China has been compared with mobile adoption in Malaysia (Ting et al., 2016), the United States (Zhang et al., 2018), and Pakistan (Akhtar et al., 2019) but not in any European country. Research on mobile payment remains interesting for academia and the business sector, and most current research is based on the extension of classical models (Tamilmani et al., 2021). Thus, our research proposes the UTAUT2 model as its fundamental basis.

### Unified Theory of Acceptance and Use of Technology 2

Venkatesh et al. (2003) reviewed eight theories used to study how and why individuals adopt new information technologies. All the reviewed theoretical models employed intention and/or use as the key dependent variable. These theories were the theory of reasoned action (Fishbein & Ajzen, 1975), the TAM (Davis, 1985), the motivational model (Davis et al., 1992), the theory of planned behavior (Ajzen, 1991), the combined TAM and theory of planned behavior (Taylor & Todd, 1995), the model of personal computer utilization (Thompson et al., 1991), the innovation diffusion theory (Moore & Benbasat, 1991; Rogers, 2003), and social cognitive theory (Bandura, 1986). The unified theory of acceptance and use of technology (UTAUT), proposed by Venkatesh, synthesizes these older technology acceptance theories, and the resulting model has been employed in various studies on technology adoption. The constructs included in UTAUT are performance expectancy, effort expectancy, social influence, and facilitating conditions moderated by gender, age, experience, and voluntariness of use. The UTAUT theory has been refined up to very recently (Dwivedi et al., 2019).

The fact that technology adoption had been studied only within organizations represented a shortcoming of all the previous models. Given the increasing necessity of investigating the determinants of technology adoption among consumers, a revision of UTAUT was published in 2012 as UTAUT2 (Venkatesh et al., 2012), which incorporated three new constructs: hedonic motivation, price value, and habit. The voluntariness of use moderator was excluded. Recently, UTAUT and UTAUT2 have been the preferred theoretical approaches to investigating mobile payment adoption (Morosan & DeFranco, 2016; Slade et al., 2014), including mobile payment adoption in the Chinese context (Hongxia et al., 2011).

### Innovation Resistance Theory

Negative factors can inhibit consumer intention to adopt innovations (Laukkanen et al., 2008; Talke & Heidenreich, 2014; Heidenreich & Kraemer, 2015). The IRT (Ram, 1987; Ram & Sheth, 1989) states that innovation means “change” to consumers and that resisting change is an expected response that must be overcome at the first stages of technology adoption. The authors suggest that users resist innovation because it represents possible changes to a satisfactory status quo or because it conflicts with the consumer’s belief structure. They divide the barriers to innovation into functional and psychological barriers. The functional barriers include usage, value, and risk, while the psychological barriers include image and tradition.

Resistance is a natural response to innovations that provoke changes to lifestyles and previous behaviors (Ram & Sheth, 1989). Organizations that offer innovative solutions urgently need to better understand the phenomenon of resistance to innovation as a crucial factor in failure or success when innovations are introduced to the market (Heidenreich & Kraemer, 2016). Resistance to adoption can be divided into passive and active resistance (Heidenreich & Handrich, 2015). Active resistance responds to the characteristics of innovations, with the barriers to adoption arising from behavioral contradictions associated with the use, costs, and risks perceived in adopting an innovation. The functional barriers proposed by the IRT are suitable for studying active resistance. By contrast, passive resistance (associated with conflicts related to existing beliefs) can be studied by considering the psychological factors of image and tradition barriers (Yu & Chantatub, 2015). The breadth of the IRT makes it suitable for the research proposed in this study (Kaur et al., 2020).

Several studies have examined the importance of barriers to the adoption of technological innovations. IRT has been adopted as a sole theoretical model for empirical investigation (Borraz-Mora et al., 2017) and also combined with other, complementary theoretical approaches (Lian & Yen, 2014; Oktavianus et al., 2017; Moorthy et al., 2017). Only recently has IRT been employed as the theoretical basis of mobile payment adoption, so studies seeking evidence on consumer resistance remain scarce (Lin et al., 2019; Kaur et al., 2020).

## Research Model and Hypotheses Development

The proposed research model assumes that both drivers and barriers to adoption impact the adoption of mobile payment and that they complement each other. A model comparing the adoption of technology in two countries with different adoption rates needs to consider both positive and negative factors to explain the phenomenon and provide more relevant results than a model that considers only one type of factor. Consequently, this study’s research model integrates IRT and the UTAUT2. Four reasons support the decision to integrate the two models: (a) to increase our knowledge of consumer adoption, it is necessary to use broader theoretical models rather than a single adoption model (Dahlberg et al., 2015); (b) no single model is sufficiently comprehensive to cover all aspects of new technology adoption behavior (Shen et al., 2010); (c) an integrative perspective provides a fuller account of a relationship’s causal mechanism as well as unique insights that cannot be obtained from a single theory–driven model (Jackson et al., 2013); and (d) an integrated model ensures greater significance and predictability of results (Oliveira et al., 2016). The UTAUT2 and IRT have recently been integrated in other studies in fields close to mobile payment adoption; Soh et al. (2020) investigated the factors affecting perception, acceptance, and willingness among older adults in Malaysia in regard to online shopping, and Sivathanu (2019) studied the adoption of digital payment systems in India.

In the proposed research model, the UTAUT2 and IRT analyze, respectively, the drivers and barriers to adoption. Based on the UTAUT2, the proposed drivers include performance expectancy (PE), social influence (SI), facilitating conditions (FCs), hedonic motivation (HM), and price value (PV). The habit construct is not included in the model, as mobile payment systems have not been on the Italian market long enough and are not sufficiently widespread to generate a habit (Oliveira et al., 2016). Additionally, the UTAUT2’s effort expectancy (EE) and IRT’s usage barrier have a common origin, namely, the concepts of the complexity and ease of use of innovations (Venkatesh et al., 2003; Laukkanen et al., 2008). Therefore, only EE is included, as it belongs to the more empirically tested model. Based on IRT, the proposed barriers include the value barrier (VB), risk barrier (RB), tradition barrier (TB), and image barrier (IB). The first two barriers are functional barriers (VB, RB), whereas the other two (TB, IB) are psychological barriers. A reverse scale is used for some of the indicators of these variables. Venkatesh et al. (2012) used the behavioral intention (BI) to adopt mobile internet as a first-order dependent variable, whereas Laukkanen (2016) used the consumer decision to adopt mobile banking. Given the similar meaning of the two dependent variables, all the constructs of the proposed research model are defined as positive or negative determinants of BI.

The first six hypotheses derive from the UTAUT2 and reflect factors with a positive effect (drivers) on the BI to adopt mobile payment. The subsequent four hypotheses stem from IRT and deal with factors with a negative effect (barriers) on the BI to adopt mobile payment. In addition, the moderating effect of culture (using Hofstede’s dimensions) is proposed to analyze differences in intention between Italian and Chinese users.

**Fig. 1:**
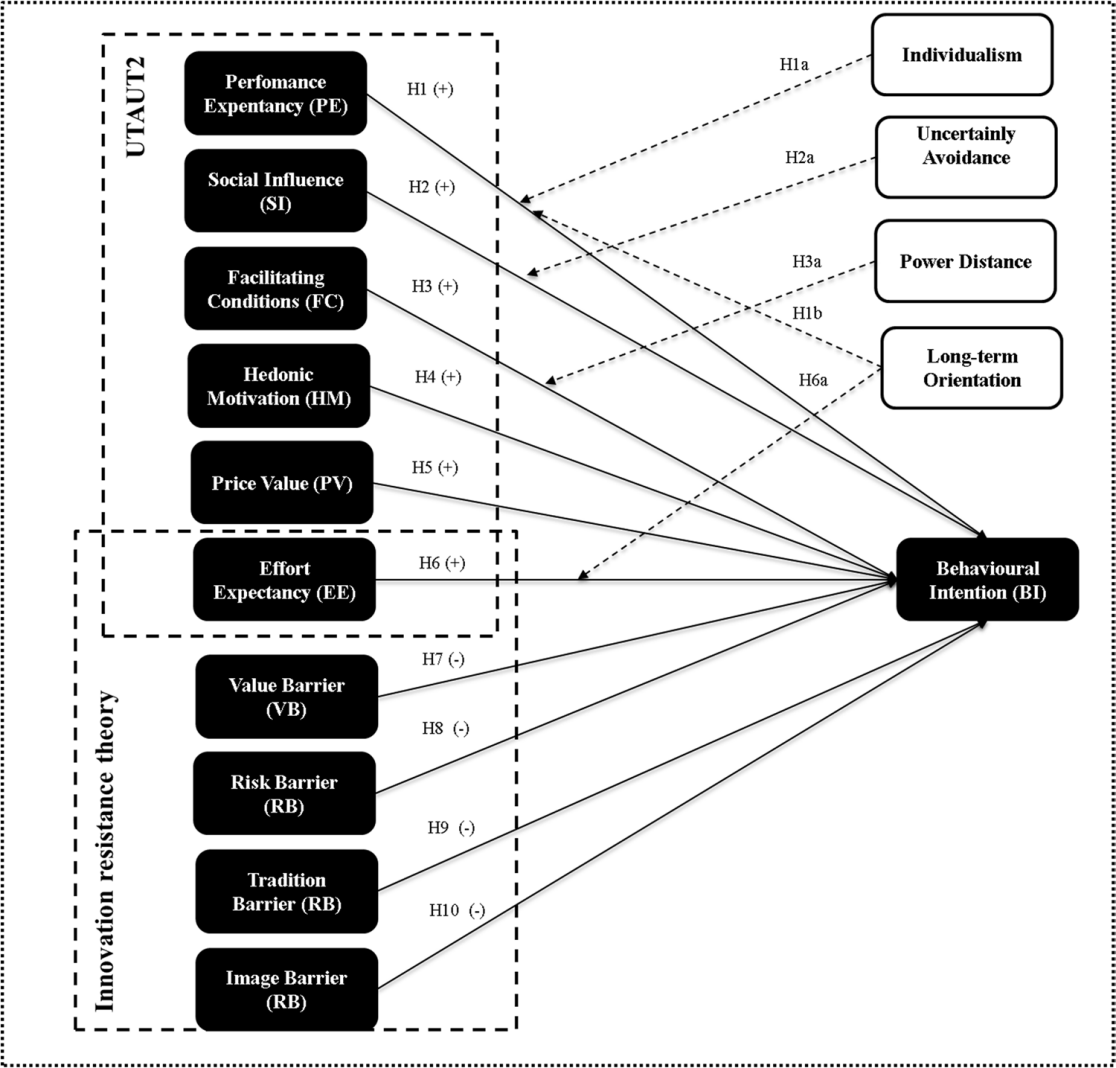
Proposed behavioral model

The research model is illustrated in Fig. 1.

### UTAUT2 Hypotheses

PE is “the degree to which using technology will provide benefits to consumers in performing certain activities” (Venkatesh et al., 2012, p. 159), and it is likely to exert one of the stronger influences on BI (Venkatesh & Zhang, 2010). The utilitarian benefits offered by mobile payment are expected to be important drivers of adoption, as they offer a convenient way to complete financial transactions with virtually no spatial restrictions. The positive effect of PE on BI has been confirmed in research in the context of mobile payment (Slade et al., 2014; Teo et al., 2015; Thakur, 2013; Wang & Yi, 2012).

- H1. PE positively influences the BI to adopt mobile payment.

SI indicates “the extent to which consumers perceive that important others (e.g., family and friends) believe they should use a particular technology” (Venkatesh et al., 2012, p. 159). Although SI has been widely tested in the context of mobile payment, the obtained results are mixed. Its impact on BI has been supported (Slade et al., 2014; Tan et al., 2014; Yang, 2012) as well as rejected (Teo et al., 2015; Wang & Yi, 2012) across diverse empirical studies. Here, we assume that, when consumers recognize that mobile payment is important to their acquaintances and when their opinion is positive, consumers are encouraged to adopt mobile payment.

- H2. SI positively influences the BI to adopt mobile payment.

FCs refer to “consumers’ perceptions of the resources and support available to perform a behavior” (Venkatesh et al., 2012, p. 159). Using mobile payment requires infrastructure, such as reliable internet coverage, and personal attributes, such as a feeling of confidence when using a smartphone for payments. The BI to adopt mobile payment increases within a supporting operational infrastructure, and research on mobile payment supports the significant effects of FCs (Teo et al., 2015; Yang, 2010).

- H3. FCs positively influence the BI to adopt mobile payment.

HM is understood as “the fun or pleasure derived from using technology” (Venkatesh et al., 2012, p. 159). As a new form of payment, mobile payment has the potential to be enjoyable to users (Oliveira et al., 2016). If it is, in fact, enjoyable, they are more likely to adopt it.

- H4. HM positively influences the BI to adopt mobile payment.

PV is defined as “consumers’ cognitive tradeoff between the perceived benefits of the applications and the monetary cost for using them” (Venkatesh et al., 2012, p. 159). It may include factors such as device cost, mobile carrier costs, and transaction fees (Baptista & Oliveira, 2015). Adoption increases when the perceived benefits of innovations are greater and when the perceived monetary value is low.

- H5. PV positively influences the BI to adopt mobile payment.

EE is the “degree of ease associated with consumers’ use of technology” (Venkatesh et al., 2012, p. 159) and is strongly connected with the perceived ease-of-use concept in the TAM, from which also stems the usage barrier construct of IRT (Laukkanen, 2016). As mobile payment demands less physical and mental effort than traditional methods of payment, the degree of perceived ease associated with mobile payment is likely to affect BI (Teo et al., 2015).

- H6. EE positively influences the BI to adopt mobile payment.

### Innovation Resistance Theory Hypotheses

The VB requires that innovations offer superior performance in relation to price than existing alternatives for consumers to change their behavior (Laukkanen, 2016). Unless an innovation offers a higher value than existing products, customers have no reason to change (Ram & Sheth, 1989). Hence, consumers adopt mobile payment if it provides advantages over other methods, such as cash or bank cards. The VB has been found to impact innovation resistance to adopting digital payment (Sivathanu, 2019) and to negatively affect the willingness to engage in online shopping (Soh et al., 2020).

- H7. The VB negatively influences the BI to adopt mobile payment.

The RB refers to the degree of risk inherent in innovations, such as financial, psychological, physical, or social risk (Laukkanen, 2016). Users associate various risks with payment transactions, such as security and privacy concerns, risks inherent in the online channel (Forsythe and Shi, 2003; Kuisma et al., 2007), and confidentiality concerns about the PIN and authentication mechanisms (Liao & Cheung, 2002; Thakur & Srivastava, 2014). The more mobile payment systems are perceived as risky, the less likely consumers are to adopt them.

- H8. The RB negatively influences the BI to adopt mobile payment.

Kleijnen et al. (2009) distinguish between tradition and norms in referring to the societally related context and usage patterns associated with personal routines and habits. The TB comes into play when an innovation conflicts with consumers’ existing values and past experiences as well as social norms (Laukkanen, 2016). If mobile payment conflicts with any of these, consumer adoption becomes less likely (Sivathanu, 2019; Soh et al., 2020).

- H9. The TB negatively influences the BI to adopt mobile payment.

IBs are evoked, for example, by the product category to which the innovation belongs, by the country of origin, or by the brand (Laukkanen et al., 2008). If users unfavorably associate innovations with negative images, there is a barrier to adoption (Ram & Sheth, 1989). According to Laukkanen (2016), IBs are related to the concept of technology readiness, which is a combination of beliefs and feelings related to technology in general (Ferreira et al., 2014). One recent study using IRT found no support for the effect of IBs on willingness to shop online (Soh et al., 2020). However, the IB effect was found to be significant in regard to innovation resistance to using digital payment (Sivathanu, 2019). We assume that conflicts resulting from consumers’ negative associations are likely to influence the adoption of mobile payment.

- H10. The IB negatively influences the BI to adopt mobile payment.

### The Moderating Effect of National Culture on Intention to Use

**Fig. 2:**
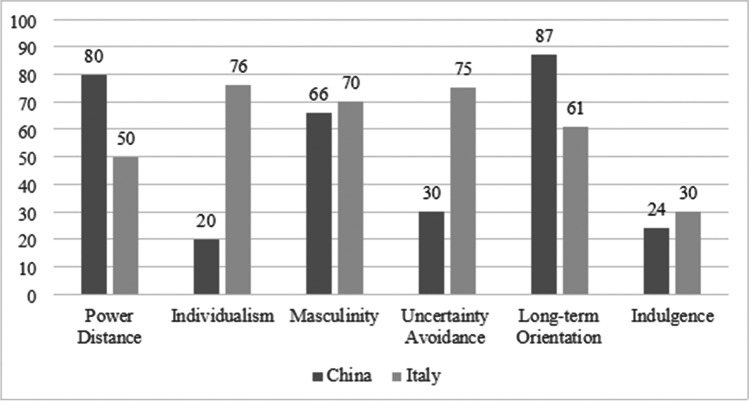
Cultural dimensions comparison between China and Italy. (Source: Hofstede, 2001)

Cultural dimensions are known influencers of consumer behavior (Hofstede et al., 2010), which may vary within six cultural dimensions (Fig. 2): (a) power distance (the acceptance of unequal power distribution in society); (b) individualism versus collectivism (the tendency to integrate into strong, cohesive groups); (c) masculinity versus femininity (the preference between male-associated qualities, such as assertiveness and material success, and female-associated ones, such as modesty and quality of life); (d) uncertainty avoidance (the fear of unknown situations); (e) long-term orientation (persistence and thrift enabling future rewards); and (f) indulgence (the tendency to seek happiness) (Hofstede, 2001; Zhang et al., 2018). The influence of culture on mobile technology adoption has been investigated in China and the USA (Zhang et al., 2018), Indonesia and Malaysia (Aji et al., 2020), and China, France, and Thailand (Dutot et al., 2019), among other places.

Although these indicators were not measured in this study’s sample, the scientific literature contemplates the use of the values published by Hofstede (Gong et al., 2007; Pavluković et al., 2017; Zhang et al., 2018). Several studies have shown that culture is crucial in new technology adoption (Bankole & Bankole, 2017), and important differences are observed in the dimensions of individualism, uncertain avoidance, power distance, and long-term orientation. It is in relation to these dimensions that we propose the hypotheses below.

The dimension of individualism involves the relationship between individuals and the collectivity that characterizes all societies (Hofstede, 2001). Individualism is typical of societies in which people’s ties among themselves are weak, whereas collectivism is present in societies in which people are integrated from birth into strong, cohesive groups. Users in individualistic cultures are more likely to adopt new purchasing technologies (Zhang et al., 2018; Chopdar & Sivakumar, 2019; Zhao & Bacao, 2020), are less affected by the opinion of their peers, and are more focused on efficiency, speed, and performance (Alcántara-Pilar et al., 2017; Srite & Karahanna, 2006). Lim et al. (2004) propose that, by contrast, users in countries with greater collectivism have a lower intention to adopt new purchasing technologies through online platforms. We compare an individualist country, Italy, with a collectivist one, China, and propose the following hypothesis:

- H1a: The relationship between PE and BI is stronger in individualistic national cultures than in collectivistic ones.

Uncertainly avoidance is defined as the extent to which people feel frightened by a situation’s uncertainty and ambiguity (Hofstede, 2001). Uncertainty affects the acceptance of a technology and its possible drivers and barriers (Sheikh et al., 2017; Al-Okaily et al., 2020; Sharma et al., 2020). Individuals in a culture with high uncertainty avoidance more strongly consider SI (as defined by the UTAUT2), whereas those in a culture with low uncertainty avoidance are less affected by SI when dealing with a new technology (Alhirz & Sajeev, 2015). Consequently, uncertainty avoidance may moderate the relationship between SI and the intention to use new mobile payment systems (Lai et al., 2016). We compare a country with high uncertainty avoidance, Italy, with one with low uncertainty avoidance, China, and propose the following hypothesis:

- H2a: The relationship between SI and BI is stronger in cultures with a higher level of uncertainly avoidance than in those with a lower value.

Distance to power is the degree to which people with less power in institutions and organizations accept and expect that power is distributed unequally (Hofstede et al., 2010). This cultural dimension has been studied in diverse research on technology adoption (Mahfuz et al., 2016; Merhi et al., 2019; Nugroho et al., 2020). Members of societies with a high score in distance to power are more likely to accept that some individuals have more power than others, whereas member of societies with a lower score tend to prefer a more egalitarian social structure (Alcántara-Pilar, 2012). In societies with greater distance to power, the adoption of a new mobile payment system by those higher in the social hierarchy increases the likelihood that those lower in the hierarchy will accept it (Goularte & Zilber, 2019). We compare a country with high distance to power, China, with one with low distance to power, Italy, and propose the following hypothesis:

- H3a: The relationship between FCs and BI is stronger in cultures with a high level of distance to power than in those with a low distance to power.

Long-term orientation describes preferring to invest time to obtain long-term results than to obtain immediate results (Hofstede, 2001). Chinese culture has a high score in long-term orientation, and its members are expected to look for performance expectations and invest more to achieve it. If those in societies with stronger long-term orientation perceive a technology to offer better long-term performance, the effect of EE on usage intention will be greater for them than for those in short-term oriented societies (Alcántara et al., 2018; Zhang et al., 2018). We compare a country with long-term orientation, China, with one with short-term orientation, Italy, and propose the following hypothesis:

- H6a: The relationship between EE and BI will be stronger in users in long-term oriented cultures than in users in short-term oriented cultures.

In addition to the above, PE is associated with the degree to which people believe that using mobile payment will help them achieve their objectives better than existing payment systems. The PE of those in cultures with a long-term orientation is positively correlated with the adoption of an innovation. By contrast, people in short-term oriented cultures want the procedure to be easy and are less willing to try new technologies (Alcántara-Pilar et al., 2018). We propose the following hypothesis:

- H1b: The relationship between PE and BI will be stronger in societies with a long-term orientation than in societies with a short-term orientation.

## Research Methodology

### Measurement Development

We designed a questionnaire to collect the data, and the measurement scales of the research model constructs were based on previous, related studies (Laukkanen, 2016; Oliveira et al., 2016; Venkatesh et al., 2012). Ten experts reviewed the methodology and the measurement scales to ensure content validity and the appropriateness of the questions’ wording. We used 7-point Likert scales ranging from “strongly disagree” to “strongly agree” to measure the construct items.

The resulting questionnaire has three sections. After choosing the language and reading a brief introduction, the respondents were presented with questions on the measurement items of the research model, with one construct per page. Table 1 lists all the measurement items and their sources. All the questions in this section were mandatory, so the respondents could not proceed to the next page without answering all the questions on a page. For all the constructs, responses without an answer were deleted before analysis. To avoid order-effect bias, two-level randomization was applied (Perreault, 1975). The construct pages (except the page for BI) and the measurement items on each construct’s page were randomized. The second section asked questions about sociodemographic data, including age, gender, education, experience, and income. In the Chinese questionnaire, income was expressed in renminbi, with amounts close to the corresponding euro values.

**Table 1:** Measurement scales

Construct	Item	Question	Reference
Performance Expectancy	PE1	Mobile payment is a useful payment method.	Venkatesh et al., 2012; Liébana-Cabanillas et al., 2015; Abrahão et al., 2016
	PE2	Using mobile payment enable me to pay more quickly.	
	PE3	Using mobile payment helps me making payments more effectively.	
	PE4	Using mobile payment allows me to save time.	
Social Influence	SI1	People who influence my behaviour think that I should use mobile payment.	Venkatesh et al., 2012
	SI2	People who are important to me think that I should use mobile payment.	
	SI3	People whose opinions that I value prefer that I use mobile payment.	
Facilitating Conditions	FC1	I have the resources necessary to use mobile payment.	Venkatesh et al., 2012
	FC2	I have the knowledge necessary to use mobile payment.	
	FC3	I can get help from others when I have difficulties using mobile Internet.	
Hedonic Motivation	HM1	Using mobile payment is fun.	Venkatesh et al., 2012
	HM2	Using mobile payment is enjoyable.	
	HM3	Using mobile payment is very entertaining.	
Price Value	PV1	Mobile payment is reasonably priced	Venkatesh et al., 2012
	PV2	Mobile payment services are a good value for the money.	
	PV3	At the current price, mobile payment provides a good value.	
Effort Expectancy	EE1	Learning how to use mobile payment is easy for me.	Venkatesh et al., 2012
	EE2	My interaction with mobile payment is clear and understandable.	
	EE3	I find mobile payment easy to use.	
	EE4	It is easy for me to become skilful at using mobile payment.	
Value Barrier	VB1	In my opinion, mobile payment does not offer any advantage compared to handling my payments in other ways.	Laukkanen, 2016
	VB2	In my opinion, the use of mobile payment increases my ability to control my financial matters by myself. ^a^	
Risk Barrier	RB1	I fear that while I am using mobile/Internet banking services, the connection will be lost.	Laukkanen, 2016
	RB2	I fear that while I am using a mobile/Internet banking service, I might tap out the information of the bill wrongly.	
	RB3	I fear that the list of PIN codes may be lost and end up in the wrong hands.	Laukkanen, 2016
Tradition Barrier	TB1	I prefer paying with cash.	Mahatanankoon & Ruiz, 2007
	TB2	I think that cash gives a better feeling of my financial means.	
Image Barrier	IB1	In my opinion, new technology is often too complicated to be useful.	Laukkanen, 2016
	IB2	I have such an image that mobile payment services are difficult to use.	
Behavioural Intention	BI1	I intend to use mobile payment in the next months.	Oliveira et al., 2016
	BI2	I predict I would use mobile payment in the next months.	
	BI3	I plan to use mobile payment in the next months.	
	BI4	I will try to use mobile payment in my daily life.	
	BI5	Interacting with my financial account over mobile payment is something that I would do.	
	BI6	I would not hesitate to provide personal information to mobile payment service.	

The questionnaire was originally written in English. It was translated into Chinese and Italian by native speakers (other than the authors) and then translated back into English by a different native speaker to ensure translation equivalence and consistency (Brislin, 1970). If the original version of the questionnaire differed in meaning from the translation, necessary changes were made. This step was necessary for the Chinese translation. Subsequently, a pilot version of the questionnaire was distributed to 40 respondents from both countries to test the readability and clarity of the questions. The answers to the pilot test were not included in the final data set.

### Sampling and Data Collection

This study employed a non-probabilistic self-selection sampling method, and primary data were collected through a web-based, self-administered survey. Social networks and messaging apps were used as a starting point for distributing the survey link. Snowball sampling (Quinlan et al., 2019) was employed by encouraging participants to invite their acquaintances to take the survey. To ensure a high response rate, a lottery coupon to be spent on Amazon or Taobao was offered to respondents who completed the questionnaire (Sauermann & Roach, 2013). We obtained the data on national cultural dimensions from Hofstede’s research.

The online survey was conducted between November 2019 and mid-January 2020 for a total of 10 weeks. A total of 666 answers were gathered, 346 from Italians and 320 from Chinese participants. Of these, 505 were considered valid because no question relative to the model was left unanswered. Table 2 shows the respondents’ characteristics. Overall, the respondents had diverse ages, and more women than men answered the questionnaire.

**Table 2:** Characteristics of the sample

Category	Italy		China	
	f	%	f	%
Gender				
NA	6		14	
Female	163	60%	149	70%
Male	109	40%	64	30%
Age				
NA	16		24	
<18	2	1%	3	1%
18 – 25	82	31%	130	64%
25 – 35	45	17%	50	25%
35 – 45	21	8%	8	4%
45 – 55	64	25%	11	5%
55 – 65	42	16%	1	0%
> 65	5	2%	0	0%
Yearly income (€)				
NA	55		75	
0	23	10%	31	21%
EUR 1 – 9999	62	28%	72	48%
RMB 1- 77,000				
EUR 10,000 – 25,000	79	36%	38	25%
RMB – 77,000 - 200,000				
EUR 25,000 – 50,000	48	22%	10	7%
RMB 200,000 – 400,000				
Yearly income (€)				
ERU 50,000 – 75,000	7	3%	0	0%
RMB 400,000 600,000				
EUR 75,000 – 100,000	0	0%	0	0%
RMB 600,000 - 777,000				
ERU 100,000+	2	1%	0	0%
RMB 777,000+				
Education Level				
NA	2		4	
No Education	1	0%	0	0%
Primary Education	8	3%	1	0%
Secondary Education (High School)	104	38%	5	2%
Vocational Training	64	23%	123	55%
University (firsts cycle)	82	30%	92	41%
Postgraduate (PhD, Master)	17	6%	2	1%
Experience				
NA	1		4	
Never	59	21%	1	0%
< 3 years	172	62%	49	22%
>3 years	46	17%	173	78%

### Normality and Common Method Bias

Normality tests were performed based on the skewness and kurtosis values of each element. The skewness and kurtosis values of the elements were below the absolute values of 2 and 7, respectively, which allowed us to use maximum probability procedures, which indicated a similarity to the normal curve (Curran et al., 1996).

The Harman single factor test was used to examine the effect of the bias of the common method (CMB). If a single factor has a total variance of greater than 50%, the CMB is likely to influence the data and thus the empirical results (Podsakoff et al., 2003). In our study, the total variance for a single factor was 42.18%. When the full set of factors was present in the model, 57.82% of the variance was explained. This suggests that a CMB was unlikely to exist (Molinillo et al., 2019).

### Data Analysis Procedures

The analysis of the collected data was based on the structural equation model (SEM). This study used a variance-based PLS-SEM technique because: (a) the research objective was theory development, (b) the structural model was complex, (c) the sample was relatively small, and (d) a variance-based PLS-SEM requires modest assumptions on the distribution of data. SmartPLS software (v. 3.2.8) was used for the analysis (Ringle et al., 2015).

## Results

### Measurement Model: Reliability and Validity

To verify the suitability of the measurement scales, we applied various forms of analysis: reliability, validity, exploratory (using the SPSS 15.0 program), and confirmatory (using AMOS 18 software).

This research followed the two-step approach recommended by Anderson and Gerbing (1988). First, the measurement model was tested for reliability and validity, and, second, the structural model was analyzed. The measurement model tested whether the indicators correctly measured the latent variables to which they were bound. Next, the structural model tested the relationship between the exogenous factors (PE, SI, FCs, HM, PV, EE, VB, RB, TB, IB) and the endogenous factor (BI) in the research model. Measurement and structural models were first run for the two countries pooled together. Next, differences between the countries were outlined through multi-group analysis (MGA).

We then performed an exploratory factor analysis of the principal components to assess the scales’ degree of unidimensionality (Appendix 1). The analysis proved suitable for the variables under study given that: (a) the proportion of variance of all the variables (based on the Kaiser-Meyer-Olkin coefficient) always exceeded the value of 0.5, indicating sampling adequacy; (b) Bartlett’s test of sphericity showed a significance or p-value of 0.000, thus rejecting the null hypothesis of no difference between the correlation matrix and the identity matrix; and (c) the correlation coefficients of the anti-image correlation matrix of the main diagonal presented lower values (Liébana-Cabanillas et al., 2014b).

The scales’ reliability was assessed by the Cronbach’s alpha indicator (see Table 3), with 0.7 as the reference value. To test the convergent and divergent validity of the scales, a confirmatory factor analysis was performed. In this analysis, the items that contributed least to the explanatory power of the model were eliminated (*R*^2^ > 0.5). Convergent validity was evaluated using the factor loadings of the indicators. The coefficients were significantly different from zero, and the loadings between latent and observed variables were high in all cases (λ > 0.7) (variables BI6, FCs3, and VB2 were eliminated). Consequently, we can deduce that the latent variables adequately explain the observed variables.

**Table 3:** Indicators for the evaluation of the measurement model

	Item	Average	Standard deviation	Skewness	Kurtosis	t-value	p-value	α	CR	AVE
BI1	0.950	0.949	0.008	-0.074	-1.062	125.891	0.000	0.951	0.963	0.839
BI2	0.935	0.935	0.010	-0.169	-1.025	96.697	0.000			
BI3	0.945	0.945	0.009	-0.138	-1.025	106.117	0.000			
BI4	0.923	0.923	0.011	-0.243	-0.962	80.534	0.000			
BI5	0.819	0.818	0.025	-0.661	-0.695	32.583	0.000			
EE1	0.941	0.941	0.010	1.130	-1.310	90.857	0.000	0.958	0.969	0.887
EE2	0.946	0.946	0.007	0.487	-1.166	129.319	0.000			
EE3	0.952	0.952	0.007	1.038	-1.322	132.970	0.000			
EE4	0.928	0.927	0.014	1.321	-1.371	65.476	0.000			
FC1	0.926	0.925	0.011	0.565	-1.128	82.076	0.000	0.842	0.927	0.863
FC2	0.932	0.932	0.009	0.372	-1.033	100.615	0.000			
HM1	0.941	0.941	0.007	-1.114	-0.142	128.403	0.000	0.907	0.941	0.842
HM2	0.910	0.910	0.009	-0.879	-0.433	96.986	0.000			
HM3	0.901	0.901	0.013	-1.030	0.048	70.542	0.000			
IB1	0.891	0.889	0.022	0.074	0.942	40.534	0.000	0.813	0.913	0.840
IB2	0.941	0.941	0.008	0.892	1.211	113.159	0.000			
PE1	0.913	0.912	0.013	1.998	-1.472	70.493	0.000	0.946	0.961	0.861
PE2	0.932	0.932	0.010	1.432	-1.405	94.586	0.000			
PE3	0.925	0.925	0.009	0.474	-1.074	99.312	0.000			
PE4	0.942	0.942	0.008	1.345	-1.397	122.151	0.000			
PV1	0.933	0.933	0.010	-0.362	-0.505	93.170	0.000	0.915	0.946	0.854
PV2	0.934	0.934	0.008	-0.231	-0.612	122.607	0.000			
PV3	0.905	0.905	0.015	-0.402	-0.596	60.713	0.000			
RB1	0.767	0.753	0.071	-1.084	0.076	10.732	0.000	0.835	0.896	0.742
RB2	0.910	0.907	0.025	-1.127	0.105	35.883	0.000			
RB3	0.901	0.898	0.024	-0.955	-0.281	37.641	0.000			
SI1	0.918	0.911	0.076	-0.705	0.575	12.091	0.000	0.922	0.950	0.865
SI2	0.931	0.926	0.041	-0.690	0.573	22.702	0.000			
SI3	0.940	0.936	0.056	-0.608	0.570	16.870	0.000			
TB1	0.947	0.948	0.008	-0.469	0.700	117.169	0.000	0.758	0.885	0.795
TB2	0.832	0.828	0.025	-1.242	0.113	33.456	0.000			
VB1	1.000	1.000	0.000	0.575	1.128			1.000	1.000	1.000

In our model, all the exogenous factors were measured through reflective indicators. In contrast to the behavior of formative indicators, changes in the latent construct were reflected in the indicators, which were represented with arrows heading toward the latent variable. By contrast, changes in the formative indicators caused a change in the latent construct, and these relationships were represented with an arrow heading toward the indicator (Hair et al., 2011). Table 3 reports the average values and standard deviation of all the measurement items.

The PLS algorithm was used to analyze the constructs’ measurement properties. To assess the reflective outer models, the following were analyzed: (a) indicator reliability, (b) construct reliability, (c) convergent validity, and (d) discriminant validity. Indicator reliability tests whether the indicators effectively reflect the latent variable and requires factor loadings to be greater than 0.7, and indicators of newly tested items with a factor loading between 0.4 and 0.7 are considered acceptable (Hulland, 1999). Construct reliability estimates a construct’s internal consistency and verifies that all the indicators measure the same construct. It is acceptable if the composite reliability and Cronbach’s α are higher than 0.7 (Bagozzi & Yi, 1988). Average variance extracted (AVE) was used to test convergent validity. The AVE should be higher than 0.5 (Bagozzi & Yi, 1988), meaning that the latent variable explains more than half of the variance of its indicators (Hair et al., 2012).

Finding a weak correlation between the two items assessing the VB, we reverted to single item measurement, which enabled us to take advantage of a sound predictive validity (Bergkvist & Rossiter, 2007). Notably, the VB meets the condition of being a concrete attribute (Rossiter, 2002). Finally, the discriminant validity was analyzed to examine the various dimensions measured by each construct. The methods used in PLS are: (a) a cross-loading analysis to determine whether the average variance shared between a dimension and its items is higher than the variance shared with the model’s other dimensions (Barclay et al., 1995); (b) the Fornell-Larcker criterion analysis, testing whether the correlations between the various dimensions are lower than the value of the square root of AVE (Fornell & Larcker, 1981); and (c) heterotrait-monotrait (HTMT) ratio analysis, measuring whether the correlations between pairs of constructs reach less than 0.9 (Henseler et al., 2015). Table 4 gives the results of the second and third methods. In the present study, the values were close to the values recommended in the scientific literature. In light of these findings, the discriminant validity of the model was considered satisfactory.

**Table 4:** Discriminant validity (square root of the AVE in bold on the main diagonale)

	BI	EE	FC	HM	IB	PE	PV	RB	SI	TB	VB
BI	**0.916**	0.702	0.618	0.564	-0.415	0.767	0.572	-0.166	0.109	-0.446	-0.402
EE	0.733	**0.942**	0.744	0.532	-0.508	0.727	0.611	-0.176	-0.018	-0.365	-0.339
FC	0.690	0.827	**0.929**	0.386	-0.384	0.614	0.530	-0.189	0.043	-0.281	-0.288
HM	0.596	0.560	0.434	**0.918**	-0.204	0.560	0.486	-0.035	0.154	-0.240	-0.268
IB	0.463	0.564	0.456	0.226	**0.916**	-0.386	-0.234	0.463	0.287	0.513	0.436
PE	0.807	0.761	0.687	0.593	0.432	**0.928**	0.579	-0.046	0.083	-0.338	-0.407
PV	0.614	0.652	0.604	0.524	0.267	0.620	**0.924**	-0.119	0.190	-0.260	-0.288
RB	0.172	0.186	0.214	0.063	0.557	0.048	0.128	**0.862**	0.086	0.433	0.199
SI	0.116	0.030	0.050	0.171	0.329	0.087	0.208	0.094	**0.930**	0.129	0.189
TB	0.492	0.391	0.322	0.262	0.614	0.359	0.305	0.523	0.134	**0.891**	0.413
VB	0.412	0.346	0.314	0.272	0.479	0.418	0.300	0.215	0.196	0.445	**1.000**

Fornell-Larcker criterion (below the main diagonal) and Heterotrait-Monotrait Ratio (HTMT) (above the main diagonal)

### Evaluation of the Structural Model

First, the coefficient of multiple correlations to the square (*R*^2^) was assessed, as it reliably indicates the amount of variance of the construct that was explained by the model. Falk and Miller (1992) posit that a suitable value should be higher than or equal to 0.100. In the present study, the value of *R*^2^ with regard to BI was 0.689, so the recommended threshold was sufficiently exceeded.

Second, we examined the standardized regression weights showing the relative weight of the factors in the endogenous variables. According to Cohen (2013), values higher than 0.2 are recommended. In this research, the effect of some variables did not exceed the established threshold, although the values obtained were significant. Among the significant relationships of the model are SI, FCs, HM, EE, and TB, which presented a value below the recommended values. Falk and Miller (1992, p. 80) introduce a less exhaustive guideline and propose standardized regression weights of 0.15, whereby the predictor variable would explain at least 1.5% of the variance of a predicted variable. Chin (1998a) also considers that values between 0.1 and 0.2 may be considered as moderate influence.

Q^2^ and f^2^ were used to report predictive relevance and effect size (Hair et al., 2016). Q^2^ values above zero indicate that the model has predictive relevance. As Table 5 demonstrates, BI had adequate predictive relevance. In effect sizes (f^2^), values of 0.02–0.15, 0.15–0.35, and ≥0.35 indicate that an exogenous latent variable has a small, medium, or large effect, respectively (Chin, 1998a). Again, SI, FCs, HM, EE, and TB had a small-to-medium impact on BI, and PE had a medium impact on BI.

**Table 5:** Assessment (significant parameter estimates in bold) of the structural model (bootstrapping = 5,000)

	Coefficient	Path Coefficient	t-value	p-value	Hypothesis	f ^2^	Q^2^	R^2^	SRMR
H1	**PE → BI**	0.408	7.375	**0.000**	**Supported**	0.196			
H2	**SI → BI**	0.079	2.860	**0.004**	**Supported**	0.016			
H3	**FC → BI**	0.122	2.856	**0.004**	**Supported**	0.020			
H4	**HM → BI**	0.126	3.581	**0.000**	**Supported**	0.031			
H5	**PV → BI**	0.056	1.415	0.157	Not Supported	0.005			
H6	**EE → BI**	0.127	2.000	**0.046**	**Supported**	0.014			
H7	**VB → BI**	-0.045	1.443	0.149	Not Supported	0.004			
H8	**RB → BI**	-0.009	0.290	0.772	Not Supported	0.000			
H9	**TB → BI**	-0.156	4.186	**0.000**	**Supported**	0.049			
H10	**IB → BI**	-0.026	0.725	0.468	Not Supported	0.001			
	**BI**						0.538	0.689	
	**SRMR**								0.03

**Fig. 3:**
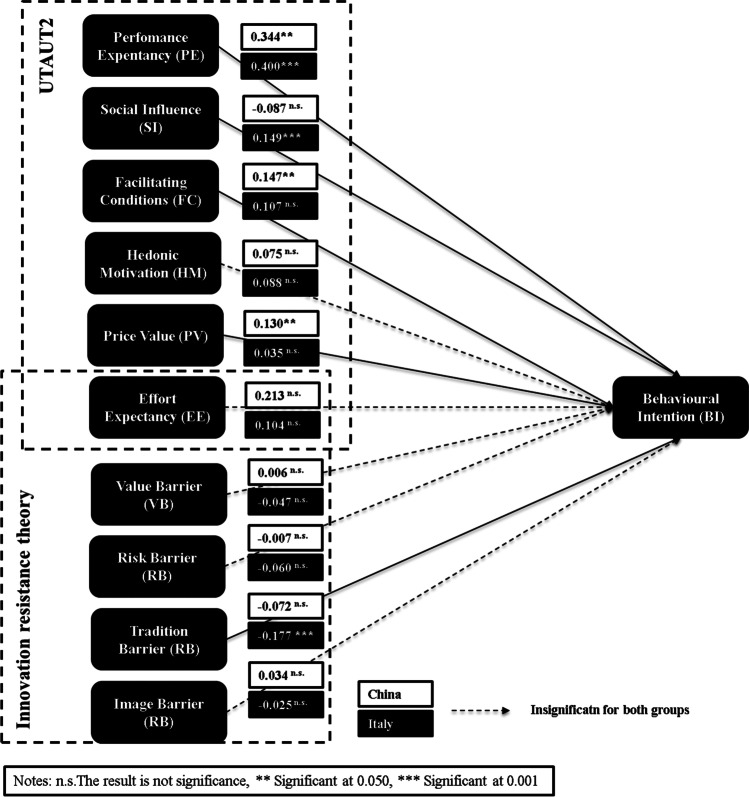
Results of the testing of the hypotheses

Finally, the value of the standardized root mean square residual (SRMR) ratio (Henseler et al., 2015) was used to compare the difference between the observed correlation and the predicted correlation as an indicator of model fit. A value below 0.08 is considered acceptable. The model in this research yielded a value close to that threshold (SRMR = 0.03). Therefore, the fit of the proposed model was considered to be adequate (see Fig. 3).

After the global model was analyzed and we had verified whether there were differences at the level of the indicators in terms of the moderating variables, we checked whether those differences also existed at the structural level. To do so, we conducted an MGA (Henseler et al., 2009; Sarstedt et al., 2011; Higueras-Castillo et al., 2019). To this end, the sample was divided into two groups by nationality (Italy = 278; China = 227). Specifically, for each variable, the path coefficients of the two resulting structural models were compared, and we evaluated whether significant differences existed using a Student’s *t*-test for independent samples (Goodman & Blum, 1996; Molinillo et al., 2021).

As a preliminary step, we explored the measurement invariance between the groups, following the three steps of the measurement of composite model invariance (MICOM) (Henseler et al., 2016): (a) configural invariance, (b) compositional invariance, and (c) the equality of composite mean values and variances. To perform the second and third steps, we conducted a non-parametric test with 5,000 permutations. The MICOM analysis of the respondents’ country variable found no significant differences in compositional scores between the two groups, but the third step yielded significant differences in the SI and BI variable measurements. Therefore, the MICOM results show a partial invariance of the measurement that was sufficient to merit applying MGA with the aim of comparing the groups’ path coefficients (Henseler et al., 2016; Frías-Jamilena et al., 2019). The results of the MGA confirm both reliability and convergent validity for both countries (see Appendices 2, 3, and 4).

As shown in Table 6, in the case of the Chinese respondents, the results reveal that only the relationships of BI with PE (H1) (β = 0.344; *p* < 0.05), FCs (H3) (β = 0.147; *p* < 0.05), and PV (H5) (β = 0.136; *p* < 0.05) were significant. In this case, the model explains 63% of the BI to adopt mobile payment, demonstrating a sound explanatory power.

By contrast, in the sample of Italian respondents, the hypotheses derived from the relationships of BI with PE (H1) (β = 0.400; *p* = 0.000), SI (H2) (β = 0.149; *p* < 0.050), and TB (H9) (β = -0.177; *p* = 0.000) were significant. For Italian respondents, the model explained 67.3% of the BI to adopt mobile payment (Chin, 1998b; Henseler et al., 2015).

**Table 6:** Multigroup analysis (significant estimates and differences in bold)

Relationship	Path China	t value (China)	p-value (China)	Path Italy	t value (Italy)	p-value (Italy)	path-diff (| China - Italy|)	t value (China vs. Italy)	p-value (China vs. Italy)
H1: PE → BI	0.344	2.410	**0.016**	0.400	6.648	**0.000**	0.056	0.387	0.699
H2: SI → BI	-0.087	1.395	0.163	0.149	3.275	**0.001**	0.236	3.130	**0.002**
H3: FC → BI	0.147	2.186	**0.029**	0.107	1.575	0.115	0.040	0.416	0.677
H4: HM → BI	0.075	1.220	0.222	0.088	1.817	0.069	0.014	0.179	0.858
H5 PV → BI	0.130	2.310	**0.021**	0.035	0.632	0.528	0.095	1.204	0.229
H6: EE → BI	0.213	1.517	0.129	0.104	1.272	0.203	0.109	0.702	0.483
H7: VB → BI	0.006	0.099	0.921	-0.047	1.188	0.235	0.053	0.779	0.436
H8: RB → BI	-0.007	0.124	0.901	-0.060	1.298	0.195	0.053	0.723	0.470
H9: TB → BI	-0.072	1.288	0.198	-0.177	3.510	**0.000**	0.104	1.390	0.165
H10: IB → BI	0.034	0.546	0.585	-0.025	0.535	0.593	0.059	0.774	0.439

To demonstrate the moderating effect of the respondents’ nationality, an MGA was performed after disaggregating the groups of respondents (China and Italy).

Hypothesis H2a, proposing that the relationship between SI and BI is moderated by uncertainly avoidance, yielded significant differences (*p* = 0.002). However, the path coefficients were significant only for Italian users (β_CHINA_ = -0.087; *p* = 0.167; β_ITALY_ = 0.149; *p* = 0.001). Furthermore, hypotheses H1a and H1b, proposing that the relationship between PE and BI is moderated, respectively, by the dimensions of individualism and long-term orientation, yielded no significant differences (H1a: *p* = 0.699; H1b: *p* = 0.699), although the path coefficients verify this relationship (β_CHINA_ = 0.344; *p* = 0.016; β_ITALY_ = 0.400; *p* < 0.001). Hypotheses H3a and H6a, proposing respectively that the relationship between FCs and BI is moderated by power distance and that the relationship between EE and BI is moderated by long-term orientation, did not yield significant differences (H3a: *p* = 0.677; H6a: *p* = 0.483), and the path coefficients did not verify the relationship.

## Main Findings

The proposed theoretical model has identified PE, SI, FCs, HM, and EE as significant antecedents of the intended use of mobile payment. In addition, the TB was the only barrier to the intention to use. In regard to the comparison between Italy and China, the proposed research model has good explanatory power, explaining 67.3% of the variation in the BI of respondents in Italy and 63% of the variation in the BI of respondents in China.

The findings reveal that PE is significant for the BI to adopt mobile payment for both Italian and Chinese respondents (H1). This result is consistent with previous studies (Oliveira et al., 2016; Chopdar et al., 2018; Hongxia et al., 2011; Venkatesh et al., 2012). This implies that respondents value the useful and advanced functions in mobile payment. SI is significantly divergent in the two countries, with a positive, significant influence for Italian respondents and a negative, non-significant effect for Chinese respondents (H2). This result supports previous findings, namely, that SI significantly differs between potential adopters and post-adopters (Yang et al., 2012). The divergent results can be explained by the fact that Italy is behind China in this technological adoption, leading people to rely more on their peers’ opinions before using the new technology. The existing literature has found both a significant, positive influence (Liébana-Cabanillas et al., 2015) and a non-significant relationship between SI and BI (Wang & Yi, 2012; Chen et al., 2019). PV’s influence on BI (H5) reflects a positive, significant relationship for Chinese respondents and no significant relationship for Italian respondents. This finding fits the divergent adoption patterns in the two countries; late adopters are usually less price sensitive (as holds in this study for the majority of Italian respondents) than early adopters (Frank et al., 2015) (represented in this study by the Chinese sample). Respondents in China evaluated mobile payment in terms of costs and benefits, probably due to the wide use of the technology. FCs significantly influence the intention to use mobile payment for respondents in the Chinese sample but not for respondents in the Italian sample (H3), a result in line with previous research (Baptista & Oliveira, 2015; Chopdar et al., 2018). HM (H4) had no significant influence in either sample, which confirms the results of some previous studies (Oliveira et al., 2016) but diverges from others (Baptista & Oliveira, 2015; Chopdar et al., 2018). HM may not be an appropriate construct for measuring the adoption of technologies—including mobile payment—that consumers use for utilitarian reasons, such as more effectively or efficiently performing a given task (Tamilmani et al., 2019). In line with the previous literature, EE (H6) had no significant influence in either sample (Baptista & Oliveira, 2015; Oliveira et al., 2016), implying that the respondents’ decision-making in both countries is not affected by mobile payment’s ease of use.

The results show differences between the two countries’ samples when analyzing the moderating effect of culture. The Chinese users exhibited a stronger relationship in the hypotheses derived from FCs, PV, EE, VB, and IB, while the Italians had a stronger relationship in the hypotheses related to PE, SI, HM, RB, and TB. Significant differences were found only in the hypothesis derived from the effect of SI on BI, with Italian respondents showing a stronger relationship than the Chinese.

Finally, in relation to the hypotheses derived from the moderating effect of the individualism, uncertainly avoidance, power distance, and long-term oriented dimensions proposed by Hofstede, only the uncertainly avoidance dimension showed significant differences between SI and BI. This relationship is stronger in cultures with a high level of uncertainty avoidance (Italy) than in those with a lower value (China) as proposed by previous research. The other three dimensions could not be empirically verified.

## Discussion

### Theoretical Contributions

Our study investigated the reasons for the mobile payment adoption gap between China and Italy by analyzing individuals’ propensity and resistance to technology adoption. We contribute to the literature first by proposing an integrated model based on UTAUT2 and IRT that aims to provide a holistic and comprehensive understanding of what triggers and inhibits mobile payment adoption (Leong et al., 2020). As discussed in Section 3, an integrated perspective provides a fuller account of the causal mechanism underlying a relationship (Jackson et al., 2013). This proved to be the case, as the *R*^2^ value of the global model with regard to BI was 0.689, distinctly higher than the values of previous studies based only on drivers or barriers to adoption, resulting in *R*^2^ values in the range of 0.5–0.6 (Slade et al., 2014, Kaur et al., 2020). We also answer Kaur et al.’s (2020) call for further investigation of the factors contributing to consumer resistance in the adoption of mobile payment solutions; those researchers proposed IRT as a theoretical framework meriting further investigation. Moreover, Tamilmani et al. (2021) conducted a systematic review of existing UTAUT2 studies, analyzing 650 articles. We close a gap in the literature by applying the UTAUT2 constructs to examine the association between the individual and demographic attributes in a cross-country context.

Our findings indicate that IRT has a weak explanatory power on BI when compared with the UTAUT2. To our knowledge, this study is the first to integrate UTAUT2 and IRT in a cross-country comparison of mobile payment adoption. Our findings demonstrate that IRT may not be suitable for analyzing the current factors of consumer resistance that inhibit the adoption of mobile payment in some contexts (e.g., cross-cultural studies). This conclusion is in line with others in the literature. As an illustration, the VB had no impact on intention to use in previous investigations of mobile banking (Laukkanen, 2016) and mobile payment (Upadhyay & Jahanyan, 2016). A possible explanation is that most mobile payment applications do not charge for processing transactions; thus, VB does not play a central role in the adoption decision (Khanra et al., 2021). RB also had no significant effect on the adoption of mobile banking (Laukkanen, 2016) or mobile payment systems (Khanra et al., 2021), and similar results were found in our samples from Italy and China. The IB findings conflict with the extant literature. Kaur et al. (2020) found no significant impact of IB, in contrast to prior studies (Moorthy et al., 2017; Oktavianus et al., 2017). The authors argue that, when the respondents’ level of technological orientation is high, IB is less likely to play a significant role in the intention to adopt mobile payment applications.

Combining the results of the cross-country comparison, three culture-related drivers are identified: (1) individualism and (2) long-term orientation enhance the effect of PE, and (3) power distance amplifies the effect of SI. The latter indicates that mobile payment users signal their superior social status by means of technical literacy. This result perfectly aligns with previous research by Steenkamp et al. (1999, p. 66), who argue that “[in] collectivistic countries, marketing communication for a new item should emphasize that it is accepted socially and allows consumers to express societal or group values.” From their perspective, the new is useful to demonstrate wealth and performance. Not surprisingly, PE also appears to be propelled by underlying cultural dimensions.

### Practical Implications

Constructs such as SI and TB behaved significantly differently in the two samples. SI had a positive influence on respondents in Italy but no influence on respondents in China. Consequently, app designers in Italy are encouraged to facilitate higher social interaction in the use of mobile payment through features such as sharing buttons that allow users to connect with friends when using mobile payment (Tan et al., 2014). Encouraging word of mouth, both offline and online, can also persuade consumers who have not yet adopted mobile payment (Kaur et al., 2020).

TB had no significant influence on Chinese respondents but significantly influenced Italian respondents, presumably because cash is now barely used in China, whereas it remains the preferred payment method in Italy. Mobile payment providers, together with public authorities when possible, should enhance the transition to mobile payment by increasing users’ awareness of the benefits of cashless payment. Additionally, to reduce the impact of the TB, mobile payment providers must identify and measure the elements that affect the routines and habits in consumers’ daily lives. One possibility is to offer free, simplified mobile payment services so that users can experience the solution and gradually change their payment pattern. Another possibility is to associate mobile payment with monetary rewards (e.g., lower charges) to encourage the use of this payment method (Leong et al., 2020).

Finally, aspects of China’s cultural, infrastructural, and digital experience can be offered to users in Italy and other countries with lower levels of mobile payment adoption. A main reason that mobile payment is so popular in China may be that it has the support and promotion of the biggest digital players: e-commerce platforms and chat apps. Over the past few years, mobile payment has become available for most daily expenses. Means of transport (taxis, trains, and airplanes) accept mobile payment, making the Chinese population comfortable with a cashless routine. Policy makers and mobile payment providers in Italy must act together to promote quality information and ways for consumers to interact with one another. Consumers who avoid uncertainty place great value on the information they receive from other consumers in their circle of contacts. Therefore, recommendation from peers, in a digital or traditional form, will reduce uncertainty and increase mobile payment adoption (Tarhini et al., 2017).

### Limitations and Future Research

One limitation relates to the sample. The Italian and Chinese samples differed in age distribution, and in both cases more women than men answered the questionnaire. Better sampling methods and dedicated agencies are available and should be used when resources allow. Second, this study adopted a cross-sectional rather than a longitudinal approach as in the original UTAUT2. Longitudinal research accounts for changes in consumers’ BI to adopt a technology over time and is particularly useful for populations that are in an early stage of adoption, such as Italy’s adoption of mobile payment. Third, this study used the data on national cultural dimensions available on Hofstede’s official website. In the future, it would be more accurate if the data on cultural dimensions were gathered through the questionnaire’s answers.

Future research should focus more closely on cross-country studies and differences in adoption patterns. The model presented in this study can be tested in various countries, especially those with different economic situations (developing countries such as Malaysia and India and developed countries such as European countries and the USA) or different cultures (for example, China and the USA). Further studies focusing on the European Union should be conducted to investigate whether drivers and barriers to adoption work similarly or differently in countries with the same currency but different cultural contexts.

Another potential direction for research is improving the proposed model, including mediator effects, such as considering the impact of age, gender, and experience with mobile payment. The model can be improved by considering other technologies (e.g., China used QR codes, whereas Italy mainly used NFCs). Previous research on the role of technology (Ramos de Luna et al., 2018) could be included in cross-country studies.

## Appendix 1

Tables 7, 8, 9, and 10.

**Table 7:** Exploratory factor analysis

	Component										
	1	2	3	4	5	6	7	8	9	10	11
PE1	0.419	0.302	0.621	0.287	0.013	0.187	0.012	-0.031	-0.155	0.158	-0.076
PE2	0.372	0.279	0.783	0.154	0.038	0.153	0.029	-0.052	-0.074	0.108	-0.062
PE3	0.392	0.341	0.653	0.219	0.052	0.264	0.024	-0.096	-0.077	0.091	-0.088
PE4	0.370	0.259	0.790	0.166	0.018	0.181	0.032	-0.042	-0.069	0.089	-0.113
SI1	0.018	0.007	0.002	0.062	0.921	0.080	0.014	0.034	0.103	0.022	0.014
SI2	0.049	-0.024	0.049	0.084	0.912	0.059	0.018	0.026	0.090	-0.036	0.023
SI3	0.076	-0.048	0.012	0.073	0.922	0.037	0.064	0.026	0.041	0.035	0.091
FC1	0.286	0.353	0.199	0.226	0.019	0.109	-0.077	0.007	-0.064	0.761	-0.070
FC2	0.288	0.552	0.192	0.167	0.019	0.071	-0.089	-0.078	-0.090	0.603	-0.026
HM1	0.240	0.155	0.143	0.163	0.062	0.873	0.033	-0.032	-0.020	0.041	-0.054
HM2	0.275	0.254	0.232	0.211	0.069	0.730	-0.049	-0.053	-0.091	0.029	-0.089
HM3	0.170	0.131	0.105	0.112	0.091	0.892	0.044	-0.042	0.007	0.061	0.001
PV1	0.210	0.200	0.136	0.850	0.118	0.154	-0.040	-0.024	-0.017	0.140	-0.042
PV2	0.246	0.265	0.176	0.805	0.093	0.157	-0.045	-0.035	-0.102	0.085	-0.086
PV3	0.228	0.239	0.169	0.789	0.092	0.179	-0.033	-0.121	0.016	0.049	-0.025
EE1	0.273	0.785	0.243	0.215	-0.043	0.177	-0.085	-0.068	-0.149	0.159	-0.008
EE2	0.355	0.758	0.227	0.228	-0.028	0.199	-0.042	-0.089	-0.128	0.121	-0.047
EE3	0.324	0.764	0.280	0.224	0.005	0.183	-0.066	-0.040	-0.137	0.133	-0.080
EE4	0.278	0.772	0.198	0.277	-0.071	0.191	-0.055	-0.051	-0.107	0.126	-0.045
VB1	-0.213	-0.089	-0.172	-0.121	0.156	-0.102	0.099	0.151	0.165	-0.061	0.886
RB1	0.000	-0.067	-0.001	-0.015	0.020	0.043	0.875	-0.006	0.040	0.044	0.104
RB2	-0.044	-0.092	-0.014	-0.049	0.063	-0.020	0.827	0.138	0.223	-0.021	0.011
RB3	-0.098	0.016	0.065	-0.029	0.011	0.011	0.827	0.258	0.099	-0.128	-0.036
TB1	-0.275	-0.204	-0.161	-0.024	0.132	-0.078	0.210	0.709	0.226	0.004	0.208
TB2	-0.119	-0.016	-0.005	-0.110	0.010	-0.047	0.225	0.894	0.071	-0.026	0.016
IB1	-0.119	-0.139	-0.112	-0.068	0.169	-0.067	0.290	0.134	0.832	-0.081	0.081
IB2	-0.232	-0.359	-0.120	-0.018	0.216	-0.002	0.245	0.177	0.682	-0.018	0.154
BI1	0.814	0.268	0.249	0.159	0.058	0.195	-0.037	-0.111	-0.085	0.115	-0.079
BI2	0.802	0.279	0.230	0.155	0.013	0.180	-0.038	-0.085	-0.169	0.122	-0.064
BI3	0.829	0.245	0.230	0.160	0.031	0.201	-0.058	-0.106	-0.097	0.084	-0.052
BI4	0.753	0.285	0.259	0.187	0.063	0.235	-0.050	-0.176	-0.042	0.062	-0.061
BI5	0.702	0.162	0.208	0.261	0.083	0.131	-0.087	-0.073	-0.035	0.131	-0.103

Principal components analysis

## Appendix 2

**Table 8:** Discriminant validity (square root of the AVE in bold on the main diagonale)

	BI	EE	FC	HM	IB	PE	PV	RB	SI	TB	VB
BI	**0.865**	0.781	0.674	0.556	0.353	0.789	0.620	0.058	0.193	0.216	0.276
EE	0.733	**0.939**	0.697	0.597	0.440	0.916	0.624	0.041	0.172	0.153	0.290
FC	0.588	0.622	**0.929**	0.459	0.271	0.677	0.569	0.062	0.147	0.065	0.232
HM	0.505	0.560	0.402	**0.895**	0.176	0.571	0.578	0.109	0.063	0.136	0.172
IB	-0.311	-0.392	-0.230	-0.149	**0.901**	0.375	0.150	0.538	0.613	0.617	0.616
PE	0.743	0.879	0.609	0.538	-0.340	**0.948**	0.583	0.036	0.136	0.179	0.280
PV	0.546	0.570	0.488	0.507	-0.127	0.533	**0.881**	0.059	0.066	0.255	0.201
RB	-0.055	-0.011	0.012	-0.095	0.410	0.011	-0.050	**0.849**	0.288	0.603	0.224
SI	-0.197	-0.165	-0.130	-0.003	0.527	-0.136	0.036	0.247	**0.917**	0.443	0.400
TB	-0.240	-0.166	-0.067	-0.101	0.523	-0.215	-0.165	0.446	0.428	**0.825**	0.401
VB	-0.264	-0.285	-0.213	-0.170	0.559	-0.275	-0.191	0.203	0.378	0.417	**1.000**

Fornell-Larcker criterion (below the main diagonal) and Heterotrait-Monotrait Ratio (HTMT) (above the main diagonal). China

## Appendix 3

**Table 9:** Discriminant validity (square root of the AVE in bold on the main diagonale)

	BI	EE	FC	HM	IB	PE	PV	RB	SI	TB	VB
BI	**0.924**	0.650	0.680	0.493	0.436	0.770	0.569	0.349	0.435	0.532	0.382
EE	0.624	**0.932**	0.893	0.393	0.565	0.631	0.627	0.405	0.209	0.414	0.261
FC	0.610	0.798	**0.926**	0.347	0.511	0.669	0.591	0.401	0.255	0.399	0.300
HM	0.475	0.381	0.313	**0.904**	0.102	0.472	0.415	0.086	0.544	0.196	0.162
IB	-0.392	-0.504	-0.428	-0.095	**0.920**	0.376	0.260	0.658	0.091	0.573	0.337
PE	0.729	0.597	0.591	0.448	-0.332	**0.908**	0.592	0.169	0.384	0.352	0.395
PV	0.542	0.596	0.527	0.400	-0.232	0.558	**0.950**	0.236	0.417	0.257	0.281
RB	-0.322	-0.364	-0.347	-0.076	0.549	-0.154	-0.214	**0.860**	0.023	0.554	0.285
SI	0.410	0.200	0.225	0.491	0.076	0.358	0.391	-0.002	**0.934**	0.108	0.029
TB	-0.474	-0.373	-0.339	-0.180	0.477	-0.316	-0.229	0.480	-0.093	**0.916**	0.410
VB	-0.373	-0.256	-0.273	-0.168	0.304	-0.381	-0.274	0.264	-0.028	0.374	**1.000**

Fornell-Larcker criterion (below the main diagonal) and Heterotrait-Monotrait Ratio (HTMT) (above the main diagonal). Italy

## Appendix 4

**Table 10:** Indicators for the evaluation of the measurement model

	Item China	Average China	Standard deviation China	t-value China	p-value China	α	CR	AVE	Item Italy	Average Italy	Standard deviation Italy	t-value Italy	p-value Italy	α	CR	AVE
BI1	0.923	0.923	0.022	41.863	0.000	0.913	0.936	0.748	0.951	0.951	0.009	108.149	0.000	0.957	0.967	0.855
BI2	0.896	0.897	0.026	34.988	0.000				0.942	0.942	0.009	99.781	0.000			
BI3	0.909	0.909	0.026	34.442	0.000				0.953	0.952	0.009	107.725	0.000			
BI4	0.886	0.886	0.031	28.893	0.000				0.921	0.920	0.015	61.968	0.000			
BI5	0.690	0.687	0.056	12.241	0.000				0.853	0.853	0.027	31.862	0.000			
EE1	0.911	0.908	0.032	28.697	0.000	0.956	0.968	0.883	0.942	0.942	0.012	76.720	0.000	0.95	0.963	0.868
EE2	0.956	0.955	0.010	92.024	0.000				0.933	0.933	0.011	83.529	0.000			
EE3	0.952	0.951	0.014	68.916	0.000				0.944	0.944	0.010	94.557	0.000			
EE4	0.938	0.937	0.017	55.296	0.000				0.908	0.907	0.022	40.848	0.000			
FC1	0.937	0.938	0.012	78.054	0.000	0.842	0.927	0.863	0.922	0.921	0.017	54.791	0.000	0.835	0.924	0.858
FC2	0.921	0.919	0.022	42.033	0.000				0.930	0.930	0.011	82.877	0.000			
HM1	0.924	0.923	0.017	53.779	0.000	0.877	0.923	0.8	0.926	0.926	0.012	80.361	0.000	0.891	0.93	0.817
HM2	0.883	0.884	0.023	37.923	0.000				0.911	0.912	0.011	85.793	0.000			
HM3	0.876	0.874	0.021	41.128	0.000				0.873	0.870	0.026	32.982	0.000			
IB1	0.858	0.847	0.064	13.366	0.000	0.779	0.896	0.812	0.902	0.899	0.024	37.466	0.000	0.821	0.917	0.847
IB2	0.943	0.945	0.018	53.196	0.000				0.938	0.938	0.013	71.487	0.000			
PE1	0.946	0.945	0.016	59.772	0.000	0.962	0.973	0.899	0.888	0.887	0.019	47.891	0.000	0.929	0.95	0.825
PE2	0.938	0.936	0.019	49.032	0.000				0.918	0.917	0.013	68.499	0.000			
PE3	0.954	0.952	0.015	62.878	0.000				0.901	0.901	0.013	67.829	0.000			
PE4	0.954	0.953	0.013	72.586	0.000				0.927	0.926	0.011	83.037	0.000			
PV1	0.897	0.897	0.021	42.975	0.000	0.855	0.912	0.776	0.961	0.960	0.007	131.031	0.000	0.945	0.965	0.902
PV2	0.902	0.903	0.015	60.685	0.000				0.954	0.953	0.008	125.111	0.000			
PV3	0.841	0.839	0.040	21.232	0.000				0.934	0.934	0.015	64.269	0.000			
RB1	0.701	0.712	0.262	2.674	0.008	0.845	0.884	0.721	0.801	0.795	0.047	16.886	0.000	0.828	0.895	0.74
RB2	0.968	0.795	0.250	3.877	0.000				0.878	0.874	0.029	30.395	0.000			
RB3	0.858	0.784	0.230	3.729	0.000				0.899	0.900	0.020	44.261	0.000			
SI1	0.947	0.947	0.059	16.057	0.000	0.91	0.941	0.841	0.935	0.935	0.012	77.662	0.000	0.927	0.954	0.873
SI2	0.899	0.886	0.068	13.267	0.000				0.931	0.930	0.015	63.682	0.000			
SI3	0.903	0.890	0.069	13.140	0.000				0.937	0.937	0.010	91.050	0.000			
TB1	0.992	0.965	0.110	8.982	0.000	0.674	0.802	0.681	0.935	0.936	0.010	90.044	0.000	0.811	0.913	0.839
TB2	0.615	0.568	0.204	3.022	0.003				0.897	0.895	0.018	48.870	0.000			
VB1	1.000	1.000	0.000			1	1	1	1.000	1.000	0.000			1	1	1

China versus Italy

## Footnotes

1
https://www.ft.com/reports/future-of-payments.

2
https://www.bloomberg.com/news/articles/2020-09-08/how-china-is-closing-in-on-its-own-digital-currency-quicktake.

3
https://www.ecb.europa.eu/pub/pdf/other/Report_on_a_digital_euro~4d7268b458.en.pdf.
